# Design, Construction, and Concept Validation of a Laboratory-Scale Two-phase Reactor to Valorize Whiskey Distillery By-products

**DOI:** 10.1021/acsengineeringau.3c00006

**Published:** 2023-05-13

**Authors:** Anga Hackula, Richard O’Shea, Jerry D. Murphy, David M. Wall

**Affiliations:** † SFI MaREI Centre for Energy, Climate and Marine, Environmental Research Institute, University College Cork, College Road, Cork T23 XE10, Ireland; ‡ Civil, Structural and Environmental Engineering, School of Engineering and Architecture, University College Cork, College Road, Cork T12 K8AF, Ireland

**Keywords:** whiskey by-products, circular economy, volatile fatty acid production, biogas, anaerobic digestion

## Abstract

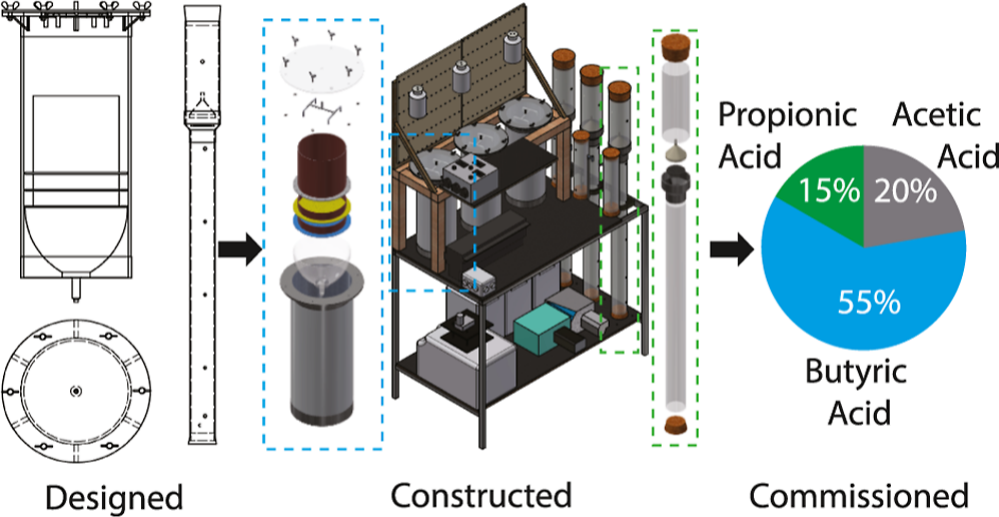

The by-products generated from the whiskey distillation process consist of organic liquids with a high chemical oxygen demand (COD) and residues with a high solid content. Low-carbon strategies that repurpose and valorize such by-products are now imperative to reduce the carbon footprint of the food and beverage industries. The operation of a two-phase anaerobic digester to produce volatile fatty acids (VFAs) and biogas may enable distilleries to transition toward a low-carbon bioeconomy. An example of such a system is a leach bed reactor connected to an expanded granular sludge bed (LBR-EGSB) which was designed, commissioned, and conceptually validated in this paper. Several design improvements progress the LBR-EGSB beyond previous reactor designs. An external gas–liquid–solid separator in the EGSB was used to capture any residual gases produced by the effluent and may reduce the amount of methane slippage and biomass washout. The implementation of a siphon-actuated leachate cup is a low-cost alternative that is less prone to actuation malfunction as compared to electrically actuated solenoid valves in previous reactor designs. Furthermore, replacing fresh water with distillery’s liquid by-products as leachate promotes a circular repurpose and reuse philosophy. The system proved to be effective in generating VFAs (10.3 g VFAs L^–1^_Leachate_), in EGSB COD removal (96%), and in producing methane-rich biogas (75%_vol_), which is higher than the values achieved by traditional anaerobic digestion systems. The LBR-EGSB could ultimately provide more by-product valorization and decarbonization opportunities than traditional anaerobic digestion systems for a whiskey distillery.

## 1 Introduction

### 1.1 Anaerobic Digestion of Whiskey By-products

Greenhouse gas emissions from burning fossil fuels have accelerated climate change’s adverse environmental and societal effects.^1^ Societal expectations have resulted in industries attempting to decarbonize and reduce their carbon footprint as part of their corporate social responsibility.^2^ Anaerobic digestion (AD) is one technology that can offer a viable pathway to reducing industry’s carbon footprint by converting biological secondary raw materials through the production of biogas, volatile fatty acids (VFAs), and biofertilisers.^3^

For optimal digestion performance, the design of the reactor should consider not only biological parameters but also technical, environmental, and economic constraints.^4^ Feedstock source, availability, inhibitors, and chemical composition will affect the digester configuration. Optimal digestion configurations seek to maximize the throughput of biological material, as quantified by the organic loading rate (OLR), while minimizing the hydraulic retention time (HRT) for biogas production.^5^ Maximizing the throughput of feedstock to the digester within the confines of biological stability maximizes the organic loading rate, minimizes the HRT, and reduces the digester volume and overall capital costs. Two-phase systems allow for additional valorization pathways through VFA production while providing for biogas production.^6,7^ VFAs can be considered a valuable commodity with a high monetary value; for example, butyric acid and propionic acid may accrue market prices of 2500 € and 2250 € per tonne, respectively.^8,9^ This additional valorization pathway could be of interest to industries which produce biological secondary raw materials, such as distillers, as it would diversify their revenue streams.

Distillery by-products are similar to brewery by-products but have more liquid derivatives.^10,11^ The liquid by-products include for thin and thick stillage; the primary solid by-product is termed draff. Draff is a lignocellulosic material due to its high lignin, cellulose, protein, and fiber content.^10^ Several previous studies have established that both brewery and distillery by-products are amenable to anaerobic digestion. The traditional continuously stirred tank reactor (CSTR) can typically accommodate feedstocks of up to 12% total solids (TS), as higher solids content could lead to stirring problems.^12^ Thus, feedstocks such as draff with high initial solids content (TS > 12% on a wet weight (ww) basis) would need to be co-digested with liquid substrates such as thin stillage (or pre-treated to reduce the overall TS content) to be used in such a system.^13^

Coupled configurations aim to overcome shortfalls in single-phase AD systems by leveraging the unique advantages of each system.^14^ An example of a two-phase AD reactor is the leach bed reactor (LBR) coupled with an expanded granular sludge bed (EGSB), referred to as the LBR-EGSB. The LBR-EGSB system splits the anaerobic digestion process into two parts: hydrolysis and acidogenesis (VFA production), which take place in the LBR, and acetogenesis and methanogenesis (biogas production), which take place in the EGSB.^7^ Dry batch AD reactors, such as the LBR, which are characterized by simple design and operation with no mechanical stirring, are potentially more suitable to digest draff due to their ability to accept high solid content feedstocks (up to 50% ww).^15^ Hydrolysis is achieved by leachate recirculation in the LBR. The liquid percolates through the organic matter and increases the leachate’s VFA content.^16^ The VFA-rich leachate can then be fed to the coupled EGSB, where biogas can be produced. The EGSB is akin to the upflow anaerobic sludge blanket (UASB). However, the EGSB has a higher aspect ratio (height-to-diameter ratio) of 20 (or more), which allows it to operate at higher upflow velocities as compared to UASBs.^5,17^ Higher upflow velocities can equate to shorter HRTs. Low upflow velocities (0.1 m h^–1^) were previously recommended for the operation of UASBs.^4^

Several studies have evaluated variations of the LBR-EGSB. The following design issues were highlighted in prior designs of similar systems: low upflow velocities as low upflow velocities decrease substrate-inoculum contact; malfunctioning of leachate actuation systems resulting in reduced hydrolysis; and back pressure within the high-rate reactor resulting in gas leaks.^4,5,7^ The LBR-UASB has been used to evaluate grass, food waste, and common reeds, achieving specific methane yields of up to 340 L CH_4_ kg^–1^ volatile solids (VS) added (ca. 243 L CH_4_ kg^–1^ chemical oxygen demand (COD) added using a conversion factor of 1.4 kg COD kg^–1^ VS).^4,18,19^ An LBR with a downstream anaerobic filter processed maize silage and reported a methane yield of 330 L CH_4_ kg^–1^ VS_added_ (ca. 236 L CH_4_ kg^–1^ COD_added_).^20^

Studies have previously analyzed coupled configurations for brewery and distillery by-products but primarily on the liquid by-products. Some coupled configurations that assessed distillery by-products include two-phase CSTRs, granular bed baffled reactors, and two-phase UASBs, which reported specific methane yields of up to 202 L CH_4_ kg^–1^ COD_added_.^21−24^ A previous study using an LBR and a high-rate reactor (granular biomass reactor) evaluated pre-treated brewers spent grains (BSGs) and reported a specific methane yield of 273 L CH_4_ kg^–1^ VS_added_ (ca. 195 L CH_4_ kg^–1^ COD_added_) but did not consider liquid brewery by-products.^25^ A two-phase EGSB reactor was used to evaluate continuous VFA production (8.4 g total VFA. L^–1^) from the BSGs hydrolysate but again did not consider the liquid by-products.^6^ Thus, there is an evident gap in literature whereby both liquid and solid whiskey by-products are processed. The authors propose the development of an LBR-EGSB to produce VFAs and biogas.

### 1.2 Objectives

This paper aims to design, construct, and provide concept validation for a two-phase LBR-EGSB to produce VFAs and biogas from whiskey by-products. This work may be considered a holistic engineering study as opposed to the biological optimization of the said system. However, concept validation will comprise short experimental trials to ensure the successful production of both VFAs and biogas.

The innovation in this paper is that it is the first paper to use the two-phase anaerobic digester, LBR-EGSB, to valorize all liquid and solid by-products (draff, thin stillage, and thick stillage) produced on-site at the distillery to produce biogas and VFAs. Furthermore, this paper will identify design improvements of the two-phase LBR-EGSB that may enhance the reactor performance beyond previously developed systems. The specific objectives of this paper are as follows:

1. design and construct a laboratory-scale leach bed reactor with an expanded granular sludge bed (LBR-EGSB) that can process distillery by-products;
2. calculate key start-up operational parameters, detail any issues encountered, and outline mitigation modifications to the design;
3. complete concept validation trials for the LBR-EGSB;
4. measure the VFA profile (from the LBRs) and the COD removal rate (from the EGSB) when digesting distillery by-products.

## 2 Materials and Methods

### 2.1 Characteristics of the Inoculum and Whiskey By-products

The granular inoculum for the EGSBs was sourced from an operational mesophilic EGSB in Co. Cork, Ireland, that processes dairy wastewater. A 3 mm mesh sieve was used to isolate the solid granules, which were subsequently used to seed the EGSBs. Before start-up, the inoculum was degassed inside the reactor for 30 days. The total solids content of the inoculum was 10.4% ww, of which 79.7% of the total solids was volatile solids. The LBRs were not inoculated.^4,7,26^

The whiskey by-products were sourced from an operational distillery and consisted of draff, thick stillage, and thin stillage, which are produced at a ratio of 1:9:10 on a wet weight basis per annum (Table 1). Due to the relatively high solids content of thick stillage (8.9% ww compared to thin stillage (TS of 2.9% ww)), a modified thick stillage was used for the experimental trials. Thick stillage was centrifuged to produce a dry fraction, termed cake maize (TS of 29.0% ww), and a liquid fraction, termed centrate (TS of 3.5% ww). The distillery currently implements this separation process. Subsequently, a modified whiskey by-product mixture (mWBM) was created based on a draff, cake maize, centrate, and thin stillage in a production ratio of 1:2:7:10 on a wet weight basis. Thus, mWBM consisted of “dry” (draff and cake maize) and “wet” (thin stillage and centrate) fractions. mWBM “wet” was further sieved with a 0.76 mm sieve to remove any solid particles prior to experiments. The production rate of by-products from an existing whiskey distillery was used to inform the relative proportions of by-products for the operation of the LBR-EGSB.

**Table 1:** Whiskey By-products Characteristics

		mWBM “dry”^a^^,^^b^		mWBM “wet”^a^^,^^c^		
		thick stillage				
	unit	draff	cake maize	centrate	thin stillage	total mWBM^a^^,^^h^
quantity	(twwta^–^^1^)^g^	31,251	58,951	218,544^h^	322,813^h^	631,559
TS^d^	(%ww)	27.4	29	3.5	2.9	6.8
VS^e^	(% TS)	96	97.2	89.3	88.8	93.7
pH	pH			3.8	4.3	4.0
COD^f^	(g L^–^^1^)			34.6	33.4	

a mWBM = whiskey by-product mixture.

b mWBM “dry” = the solid fraction of mWBM (draff and cake maize).

c mWBM “wet” = the liquid fraction of mWBM (centrate and thin stillage).

d TS = total solids on a wet weight basis.

e VS = volatile solids on a total solids basis.

f COD = chemical oxygen demand.

g twwt a^–1^ = tonne wet weight per annum.

h Theoretical value based on TS and VS annual quantities of the substrates and thick stillage (see Box S1).

### 2.2 LBR-EGSB System Components

#### 2.2.1 Overview of the LBR-EGSB Concept

**Figure 1:**
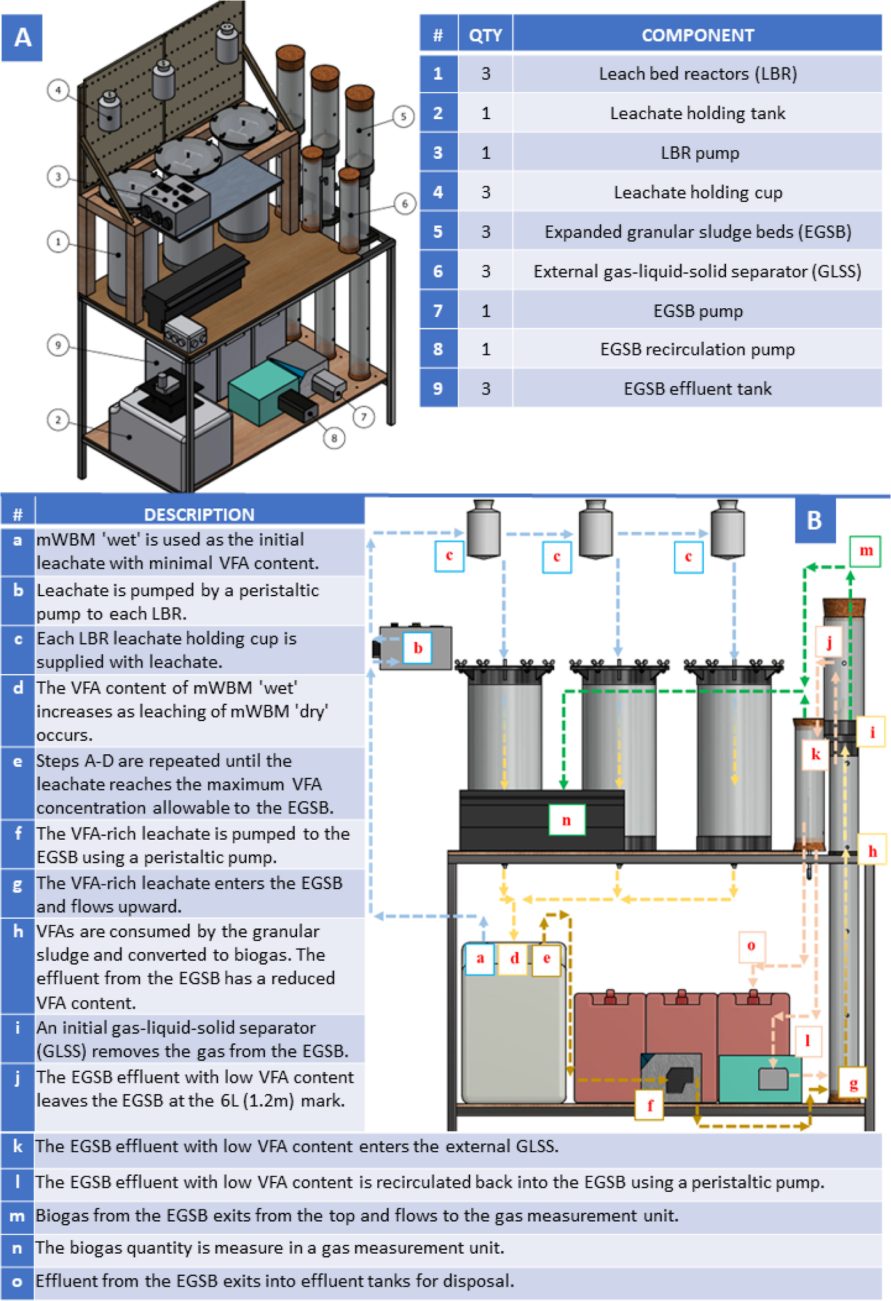
(A) LBR-EGSB components and (B) conceptual flow analysis.

The LBR-EGSB system is similar to the previous LBR-UASB configurations described in literature.^4,7,18^ The primary difference is the higher upflow velocities in the EGSB which can enable lower HRTs. The system designed herein consists of three LBRs (Figure 1A—component 1) and three EGSBs (Figure 1A—component 5). The LBRs were loaded with mWBM “dry”, and the leachate holding tank (Figure 1A—component 2) was loaded with mWBM “wet”. A peristaltic pump transferred mWBM “wet” from the leachate holding tank into the LBRs. Hydrolysis and acidogenesis occurred in the LBRs through percolation of mWBM “wet” over mWBM “dry”. During the leaching process, the VFA content of mWBM “wet” increases and is subsequently pumped with a peristaltic pump into the EGSBs, where VFAs are converted to biogas by methanogens. Figure 1 shows the detailed components and conceptual flow analysis of the LBR-EGSB.

#### 2.2.2 Leach Bed Reactors

**Figure 2:**
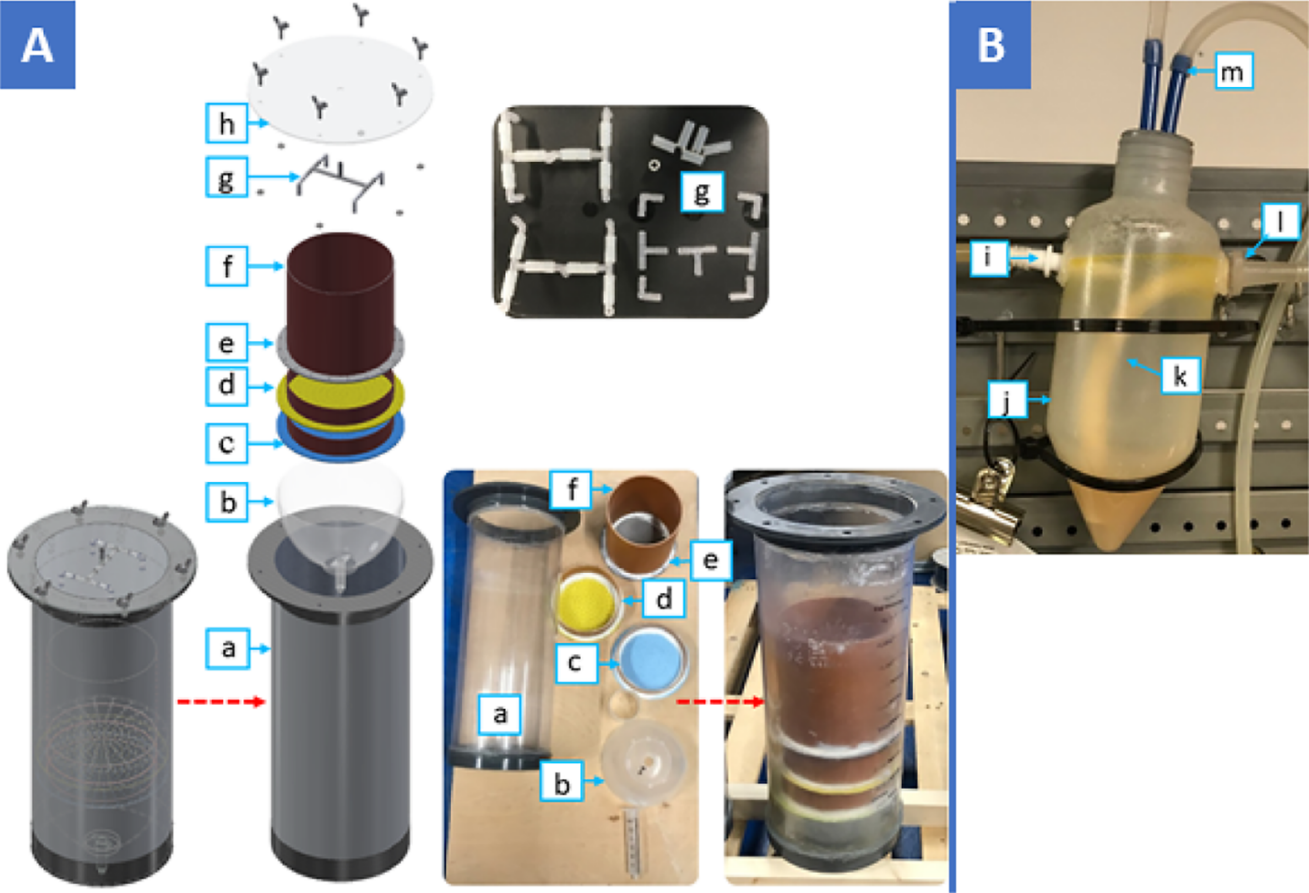
Leach bed reactor (LBR) and leachate holding cup components. (A) LBRs and the percolation system components: (a) LBR; (b) plastic bowl; (c) 1.5 mm sieve; (d) 3 mm sieve; (e) 4 mm sieve; (f) feed housing cylinder; (g) sprinkler head; and (h) LBR lid. (B) Leachate holding cup: (i) leachate inlet from the pump; (j) 250 mL container; (k) inner tube at the bottom of the leachate holding cup; (l) leachate outlet; (m) gas tube connected to the LBR lid.

Three identical LBRs of 14.9 L volume were constructed with an outer diameter of 205 mm, an internal diameter of 195 mm, and a height of 500 mm (Figure 2A—component a). The design of a single LBR allows for approximately 668 g of mWBM “dry” to be loaded. The feed housing cylinder (Figure 2A—component f) was made of PVC pipe with an inner diameter of 160 mm and a height of 150 mm. Approximately 5 L of headspace was left for the sprinkling of leachate over the feedstock, and an additional 2.5 L was left for leachate collection at the bottom of the LBR. Each LBR was wrapped with a heating mat and maintained at 37 ± 1 °C (mesophilic temperature range).

#### 2.2.3 Percolation System

A multilayer sieve was implemented to prevent solid particles from exiting the LBRs and clogging the leachate pump. The sieve hole diameters were 4, 3, and 1.5 mm, respectively, from top to bottom (Figure 2A—components c–e). In addition, mWBM “dry” was added to the LBR in a muslin cheesecloth to mitigate any washout of solid particles. The structural walls of the feed housing cylinder were made from PVC pipe, and the sieve surface was made of a 2 mm thick HDPE plastic. A 1.5 L round-bottomed plastic bowl (Figure 2A—component b) served as a funnel to direct the leachate to the outlet of the LBRs.

#### 2.2.4 Leachate Sprinkler

The leachate sprinkler (Figure 2A—component g) consisted of three T-shaped tube connectors and four L-shaped connectors made of polypropylene to distribute the leachate effectively into the LBR. The sprinkler was attached to the lid (Figure 2A—component h) of the LBRs. The overall sprinkler diameter was 120 mm.

#### 2.2.5 Leachate Holding Tank

The LBR effluent (leachate) was collected in a 25 L drum which served as the leachate holding tank. The mWBM “wet” was introduced directly into this tank. The leachate holding tank also provided a sampling point from which the leachate could be analyzed. When required, the leachate could be pumped from the holding tank to the LBRs and/or the EGSBs.

#### 2.2.6 Leachate Pump and Sprinkling System

The leachate was recirculated from the leachate holding tank (see Section 2.2.5) to the LBRs (*via* a leachate holding cup) by a bespoke variable speed peristaltic pump (45–180 L d^–1^) with three independent pump heads; each LBR received leachate independently. The sprinkling system consisted of tubing, tubing connectors, a leachate sprinkler, and the leachate holding cups (250 mL) (Figure 2B). The leachate was continuously pumped into the leachate holding cups. The leachate holding cups used a siphoning effect whereby, once the leachate reached 250 mL, the leachate emptied into the LBRs. The leachate holding cups had a gas outlet on top (Figure 2B—component m) connected to the top of the LBR headspace to allow for gas displacement in the leachate holding cup. As the hydrolysis process within the LBRs is the performance-limiting step of similar systems, malfunctioning of the leachate actuation system due to clogging, as mentioned in previous designs, would be alleviated. As previous designs used solenoid valves to initiate leachate discharge, the designed leachate holding cups are a cost-effective alternative requiring little specialized skill and are easily scalable.

#### 2.2.7 EGSB Reactors

**Figure 3:**
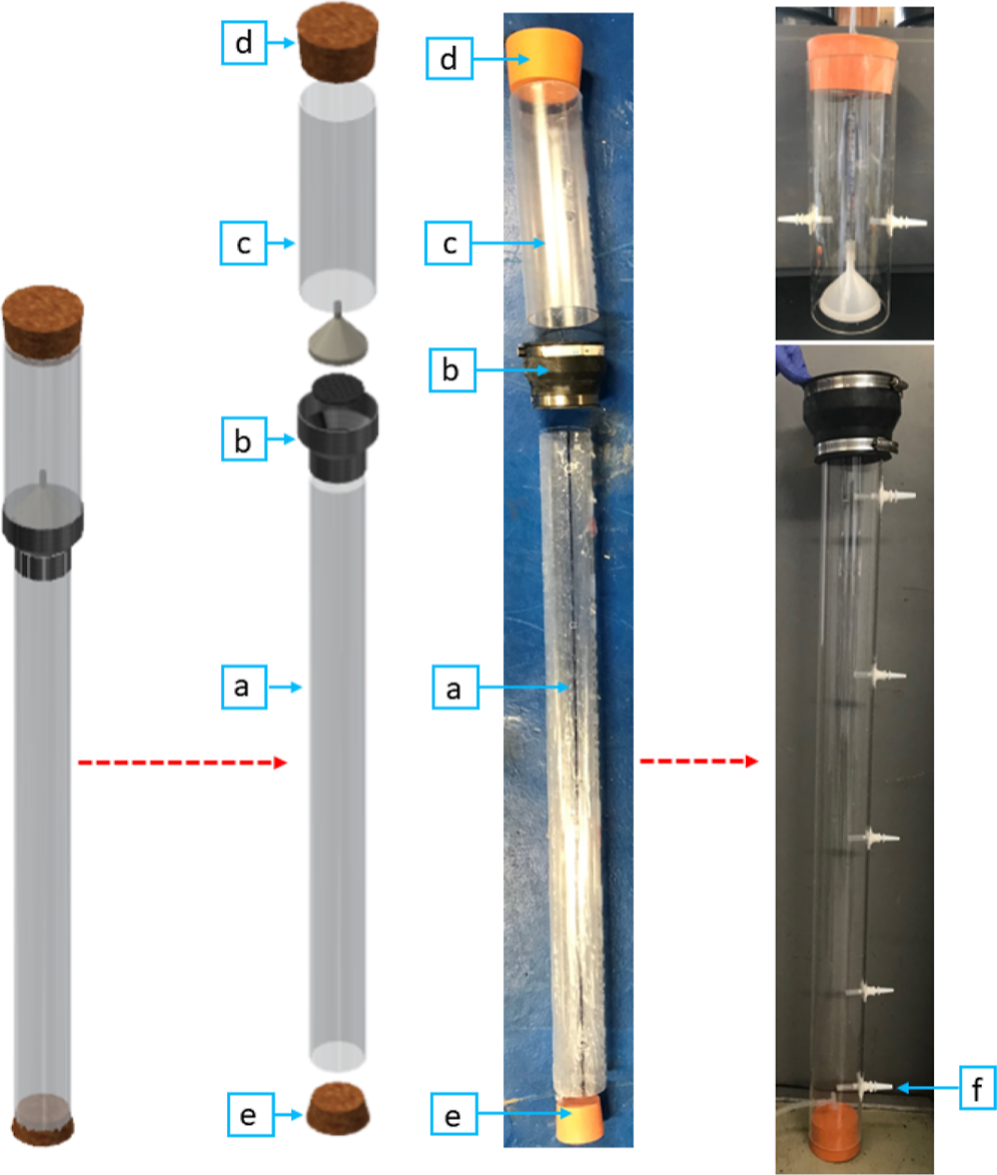
Expanded granular sludge bed (EGSB) components and assembly: (a) EGSB lower section; (b) pipe adapter coupling; (c) EGSB upper section; (d) top rubber stopper and EGSB gas outlet; (e) bottom rubber stopper; and (f) alternative recirculation inlet.

Three identical EGSBs were constructed to provide triplicate reactors for experimental trials, each with a total height of 1330 mm. Each EGSB consisted of two sections: a lower section (Figure 3a) 1000 mm in length with an internal diameter of 74 mm and an upper section (Figure 3c) 330 mm long with an internal diameter of 104 mm. The two sections were connected by a pipe adapter coupling (100 mm length) which had negligible height contribution to the overall EGSB height after assembly (Figure 3b). The increase in cross-sectional area in the upper section would serve as a diffuser and reduce the fluid velocity inside the reactor to facilitate the settlement of the granular sludge. It was hypothesized that this would reduce sludge washout at higher upflow velocities. The total volume of each EGSB was 7.1 L with a working volume of 6 L. An influent inlet was placed 50 mm from the bottom of the EGSBs to allow for space for rubber stoppers (Figure 3e). The rubber stoppers were used to seal the bottom of the reactors. Each EGSB had three sampling points located at 1 L (235 mm), 2 L (470 mm), and 3 L (705 mm) from the bottom of the reactor. The effluent point was located at ca. 1200 mm from the bottom of the reactor. The top of each EGSB was sealed using a 105 mm diameter rubber end stop (Figure 3d) with two gas outlets. The leachate was pumped into each EGSB using a variable speed Ismatec Ecoline VS-MS/CA8-6 peristaltic pump. Establishing the leachate upflow velocity in the EGSB and, thereby, the maximum pump flow rate is a crucial design parameter. A low upflow velocity (0.2 m h^–1^) was chosen for the commissioning trials to ensure little expansion of the granular sludge bed and minimal sludge washout.^5^ It was hypothesized that higher upflow velocities would be possible after acclimatization of the inoculum as inert and inactive sludge would exit the EGSB during the commissioning trial. However, the upflow velocity was still double the recommended upflow velocity (0.1 m h^–1^) of UASBs.^4^ The upflow velocity was established experimentally. A Watson Marlow 323U peristaltic pump was used to recirculate the EGSB effluent back into the EGSB through a Y-shaped connection that was also used for the leachate to maintain the required upflow velocity. The EGSBs were wrapped with heating mats (37±1 °C) to a height of 600 mm. The lower section contained a sludge bed height of 650 mm, but the EGSB was insulated with a 13 mm insulation foam. Temperature checks indicated uniform heating of the entire bed.

#### 2.2.8 Gas–Liquid–Solid Separator

**Figure 4:**
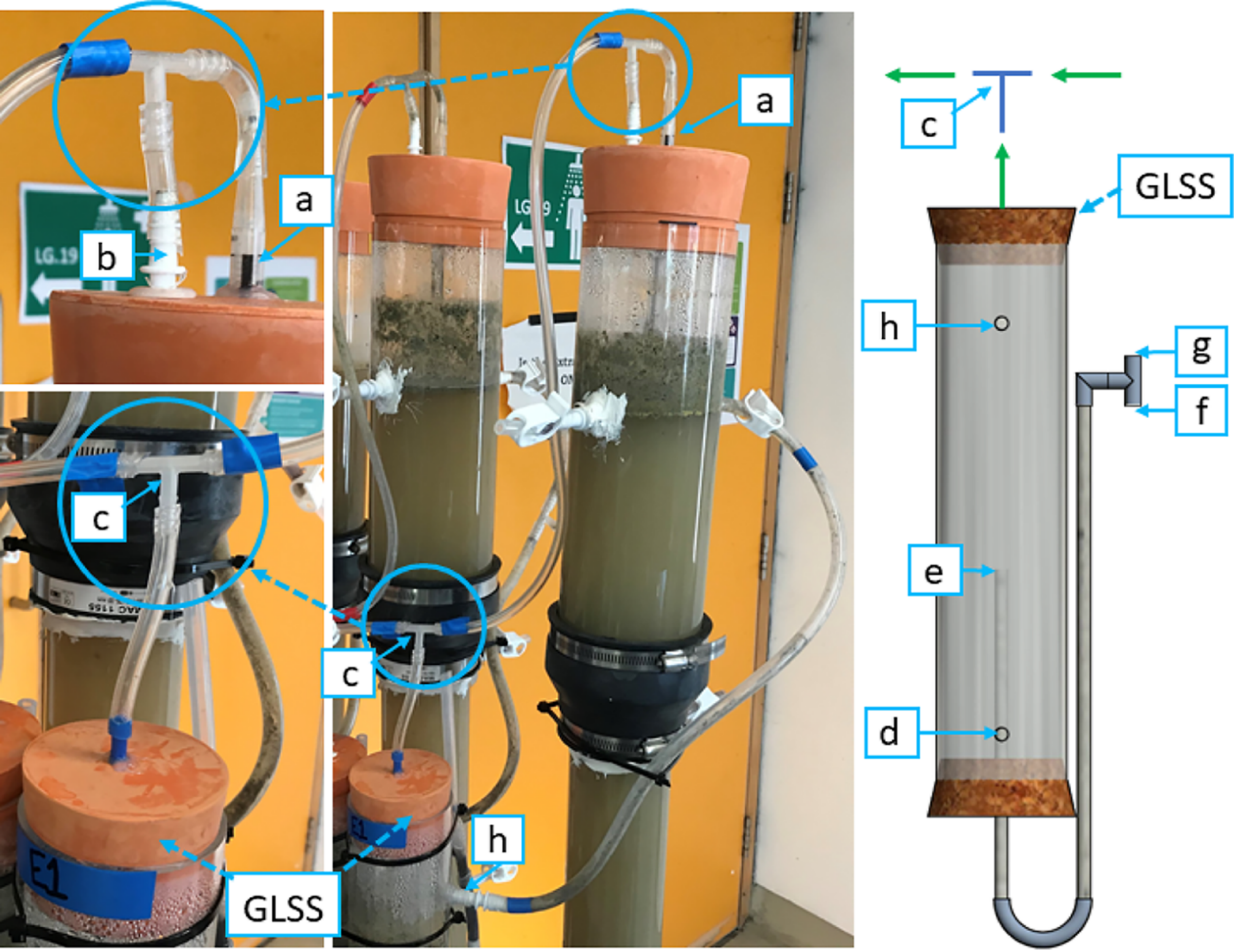
Gas–liquid–solid separator (GLSS) configuration: (a) internal GLSS outlet; (b) additional gas outlet; (c) GLSS T-shaped connection point; (d) GLSS recirculation point leads back into the expanded granular sludge bed (EGSB); (e) effluent outlet; (f) effluent tank point; (g) atmosphere; and (h) GLSS inlet from the EGSB effluent.

The gas–liquid–solid separator (GLSS) separates and collects the biogas generated from the liquids and buoyant solids within the EGSB.^27^ The EGSBs had internal and external gas–liquid–solid separators. The internal GLSS was an inverted polypropylene funnel (80 mm diameter) that was connected to the top gas outlet of the EGSB (Figure 4a). An additional gas outlet at the top of the EGSB (Figure 4b) was made for any gases that may have bypassed the internal GLSS. Once the effluent exited the EGSB, it settled momentarily in the external GLSS (Figure 4h). The external GLSS was made of a clear acrylic pipe with an inner diameter of 74 mm and a height of 300 mm. The external GLSS also served as a settling chamber for granular sludge washout. It was hypothesized that any gases that escaped with the washed-out sludge or from back pressure, as stated in previous designs, would be captured in the external GLSS. A gas outlet at the top of the external GLSS was connected to the two EGSB gas outlets (Figure 4c). The EGSB effluent was recirculated from the bottom of the external GLSS (Figure 4d). The gas outlets led to the biogas production measurement system. The effluent outlet (Figure 4e), which was approximately halfway inside the GLSS, had a U-tube connection that split into two points: one end pointed downward toward a 5 L effluent tank (Figure 4f) and the other pointed upward toward the atmosphere (Figure 4g). The GLSS also allowed for back-pressure adjustment (see Section 2.3.3). This setup was used to avoid any siphoning effects and to avoid air entering the GLSS.

#### 2.2.9 LBR-EGSB System Capacity

The total system capacity for liquids was 25 L within the leachate tank and 44 L of solids within the LBRs. The working volume in the configuration proposed for the whiskey by-products was up to 24 L for liquids and up to 28 L for solids.

### 2.3 Issues and Modifications

#### 2.3.1 Sludge Flotation

Sludge flotation caused the granular sludge bed to rise in the EGSB up to the expansion section. Sludge flotation occurred due to entrapped biogas within the hollow spaces of the sludge bed, creating a buoyant force on the granular sludge.^28^ This resulted in fluid washout (±0.3 L) and clogging. Any sludge washout during this phase was collected in the GLSS and reintroduced into the reactor. This issue was alleviated by changing the recirculation inlet to the EGSB from the same as the leachate to the lowest sample port of the EGSB, which is found on the opposite side of the EGSB influent and 50 mm higher than the influent port (see Figure 3f). It was hypothesized that this would assist in agitating the sludge bed and allow the entrapped biogas to escape.

#### 2.3.2 Settling of mWBM “Wet” Residual Solids

Residual solids within mWBM “wet” would congregate and coagulate at the bottom of the leachate holding tank and in the downstream tubing, resulting in clogging of the leachate pumping system. The leachate holding tank was subsequently fitted with a bespoke stirrer to allow for continuous agitation. This improvement may be unique to the leachate used in this system.

#### 2.3.3 Pressure Differences

**Figure 5:**
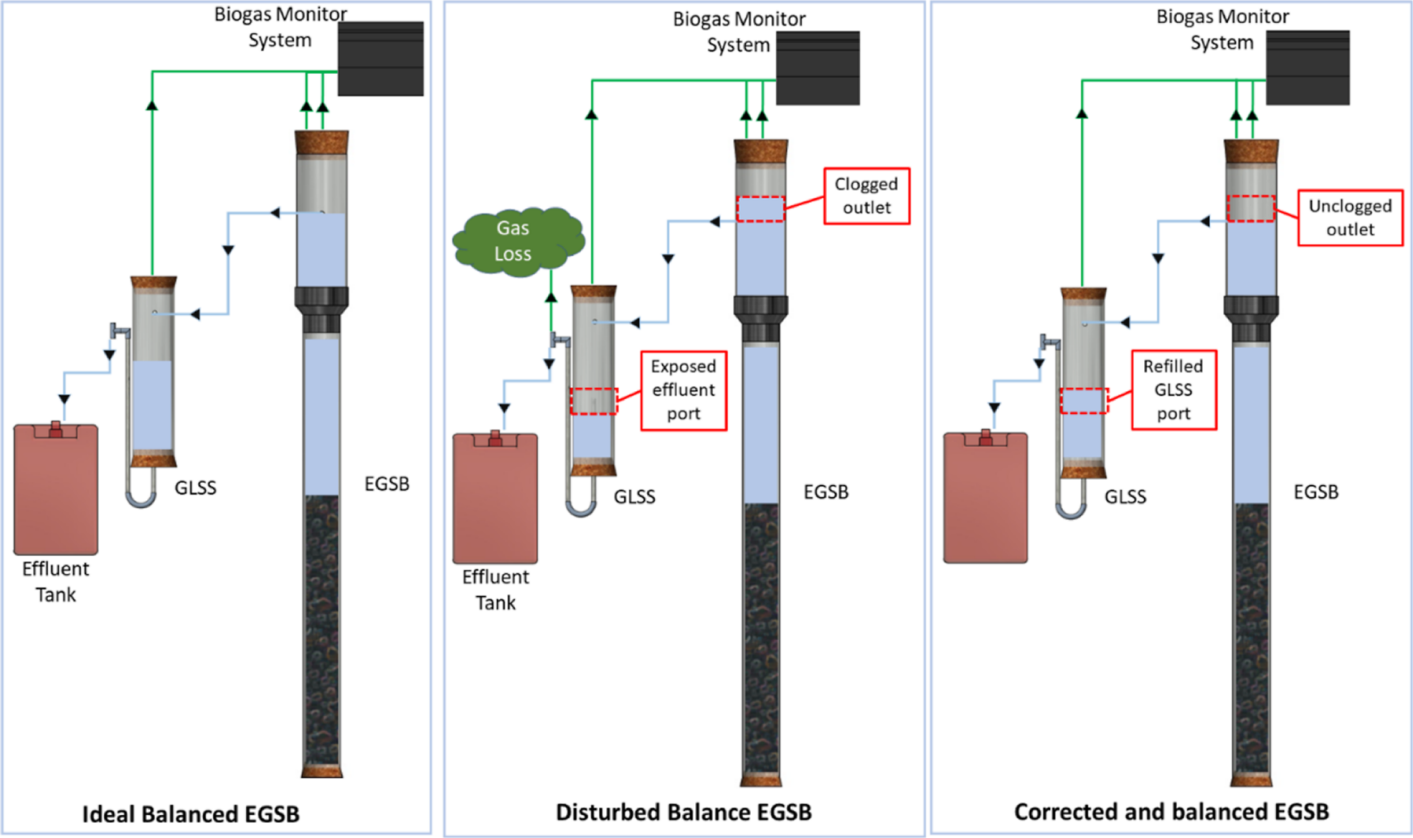
Expanded granular sludge bed (EGSB) pressure balance correction.

Pressure differences between the EGSB internal GLSS and the external GLSS had to be continuously corrected (see Figure 5). As pressure was also needed to cause the effluent to exit the GLSS, a balance was required between gas collection and effluent leaving the system; that is, equal pressure was needed to exit the system as was required to tip the gas meter. Clogging within the EGSB would result in the EGSB effluent rising beyond the effluent outlet and reducing the headspace of the EGSB. This would disturb the EGSB’s pressure “sweet spot” to register gas production. The effluent port in the external GLSS would be exposed, resulting in the biogas exiting the system *via* the external GLSS instead of being measured (Figure 5). This issue was addressed by replenishing the external GLSS liquid level with EGSB effluent from the EGSB effluent tank. The long-term solution was to separate the gas outlet of the GLSS from the main EGSB and include a larger U-tube bend between the GLSS and EGSB.

### 2.4 Calculating the Maximum Organic Loading Rate of the EGSB

The maximum COD loading capacity of the EGSB is a critical design parameter for biogas production in an LBR-EGSB system.^18^ To maximize biogas production from the EGSB, ideally, the maximum COD capacity should be fed to the reactor; this is based on the quantity of inoculum and the EGSB maximum upflow velocity (Box 1). The COD capacity of the inoculum may increase with time as the inoculum adapts to the conditions of the substrate as inert particles exit the reactors.^29^

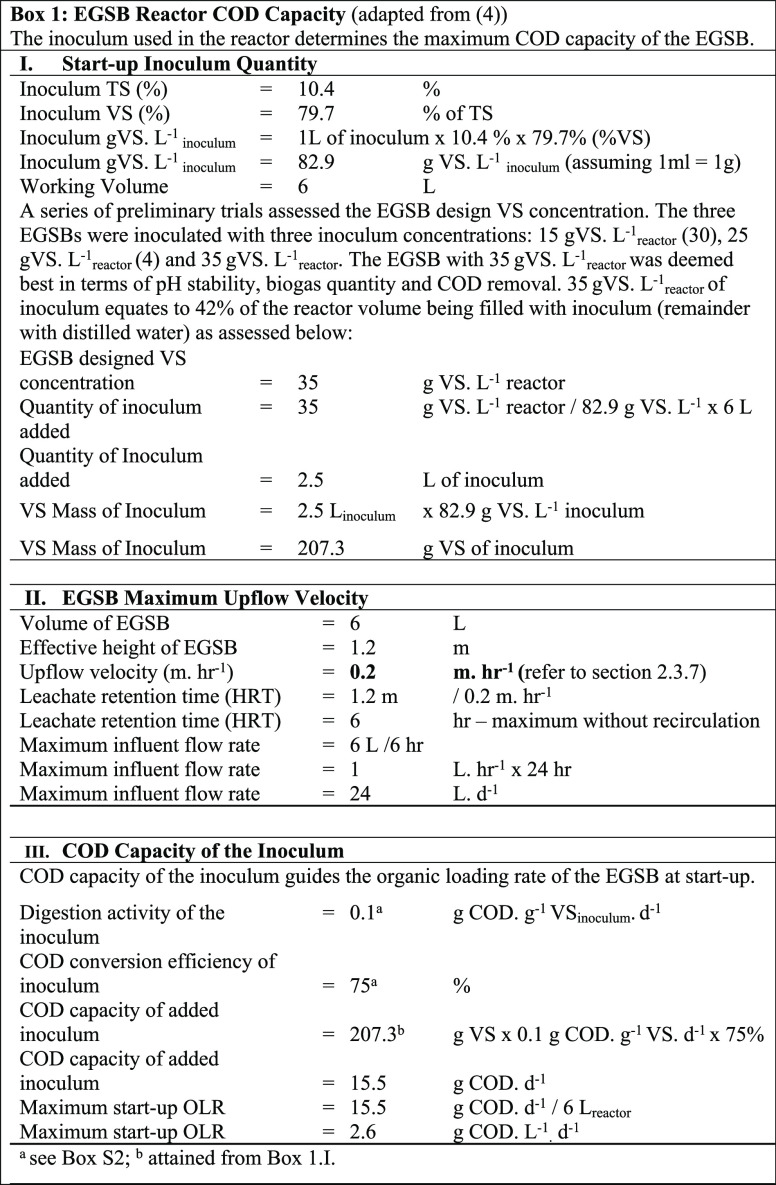



### 2.5 LBR Start-Up/Leaching Trials

The concept validation of VFA production from liquid and solid whiskey by-products within LBRs was evaluated through leaching trials. The leaching trials were phenomenological rather than an optimized biological trials. The leaching trials also served as the commissioning of the LBRs. The LBRs and EGSBs were initially commissioned separately due to the anticipated long start-up time of the EGSBs and the variable VFA concentration of the leachate.^30,40^

The LBRs were sequentially fed with 668 g of mWBM “dry” every 5 days.^19^ To mimic the liquid-to-solid ratio as produced at the distillery, approximately 4 L of mWBM “wet” was fed every 5 days directly into the leachate holding tank (see Figure 1A—component 2). The conceptual flow analysis (Figure 1B) shows the flow of the substrates within the system. The LBR leaching trials lasted 45 days. As such, each LBR was loaded three times, and mWBM “dry” was digested for 15 days. This addition was enough to facilitate high recirculation rates of the LBR of 140 L d^–1^ but did result in the pooling of the LBRs. However, this pooling effect yielded higher VFA production than using a lower recirculation rate (60 L d^–1^) (Figure S2). After loading with feedstock, the LBR and leachate holding tank were flushed with nitrogen gas for 5 min to displace air from the headspace.

### 2.6 EGSB Start-Up Trials

The EGSBs were acclimatized to whiskey by-products prior to experimental trials (Figure S3). A short experimental trial (25 days) was undertaken as a concept validation and commissioning trial. As such, optimal biological performance was not the objective of the trial. Rather, fault finding, leak detection, and design modifications were the objectives of the trial. The EGSB was connected directly to the LBR leachate holding tank, after the LBR leaching trials. As the COD of the leachate varied, the average COD over the previous 7 days was used to set the OLR to ±2.6 g COD L^–1^ d^–1^ (HRT of 6 days) for the following week. Long HRTs and low OLRs are recommended for acclimatization for wastewaters with COD concentrations greater than 5 g L^–1^.^5^ For commissioning, only the COD removal and biogas quality were assessed. The EGSBs were considered commissioned once the biogas quality was near constant, with less than 5% variation.^21^

### 2.7 Analytical Methods

The TS and VS contents of mWBM and inoculum were analyzed as per Standard Methods 2540 G.^31^ The pH of all samples was determined by a Mettler Toledo F20 pH meter. A gas chromatographer (Agilent GC 7890B) equipped with the DB-FFAP column (Ø 0.32 mm × 50 m) and the flame ionization detector was used to evaluate the VFA concentration and composition (acetic acid, butyrate acid, and propionic acid) of the LBR leachate and the EGSB effluent. VFA samples were stored at −20 °C, thawed, and centrifuged at 11,000 rpm for 10 min prior to analysis.^32^ The biogas composition of the EGSB was measured by a gas chromatographer (Agilent GC 7890B) furnished with a thermal conductivity detector and two fused silica capillary columns. Methane (CH_4_) was detected by a CP-Molsieve 5A column (25 m × 0.32 mm × 30 μm film thickness), whereas the carbon dioxide (CO_2_) content was established by a GS-CarbonPLOT column (3 m × 0.32 × 3 μm film thickness).

The COD concentration of the LBR leachate and EGSB effluent was evaluated by Hach Lange cuvette test kits (LCK 914 and LCK514) and a Hach Lange Spectrophotometer (DR 3900). COD samples were centrifuged at 11,000 rpm for 10 min and evaluated immediately.

The COD removal efficiency (COD_eff_) of the EGSB was calculated as follows^32^
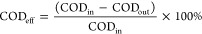
where COD_in_ is the leachate COD entering the EGSB and COD_out_ is the COD of the EGSB effluent.

Acidification of the leachate was calculated by converting the VFAs to the COD equivalent (VFAs-COD_equiv_) and dividing it by the total COD of the leachate. The COD to VFA conversion efficiency was calculated by dividing VFAs-COD_equiv_ with the COD of mWBM “wet” (33.9 g COD L^–1^).

The VS destruction efficiency (VS_dest%_) of the LBR was calculated as follows^33^
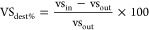
where VS_in_ is the VS mass (in grams) of mWBM “dry” before entering the LBR and VS_out_ is the VS mass of mWBM “dry” out of the LBR.

Biogas production from the LBRs was not monitored as mostly carbon dioxide was expected. Biogas gas production from the EGSB was estimated based on the system removal efficiencies: the VS destruction efficiency and the COD removal efficiency.

## 3 Results and Discussion

### 3.1 Trends in Reactor Stability

**Figure 6:**
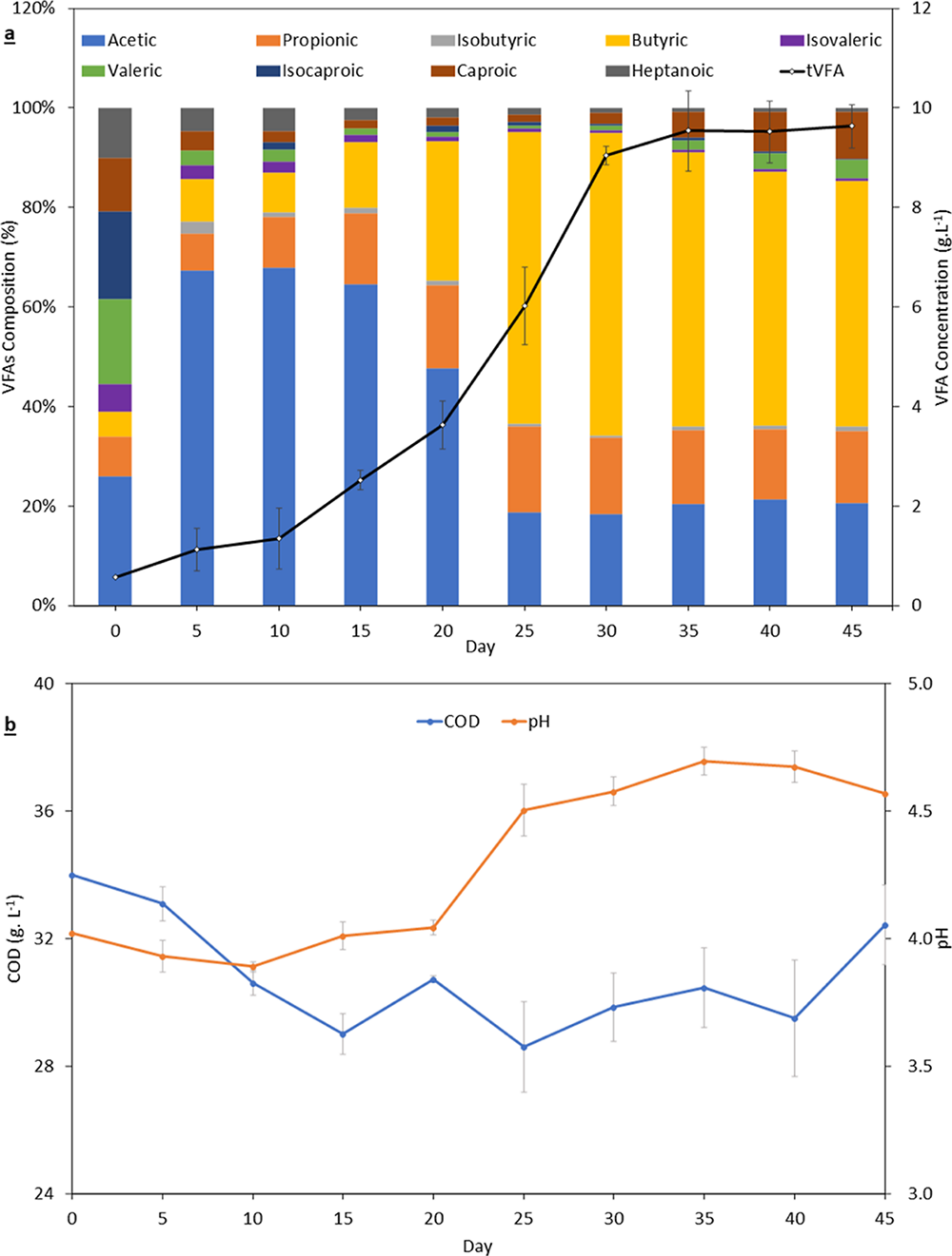
(a) Leachate volatile fatty acid (VFA) concentrations and composition, and (b) leachate chemical oxygen demand (COD) and pH from the leaching trials.

**Figure 7:**
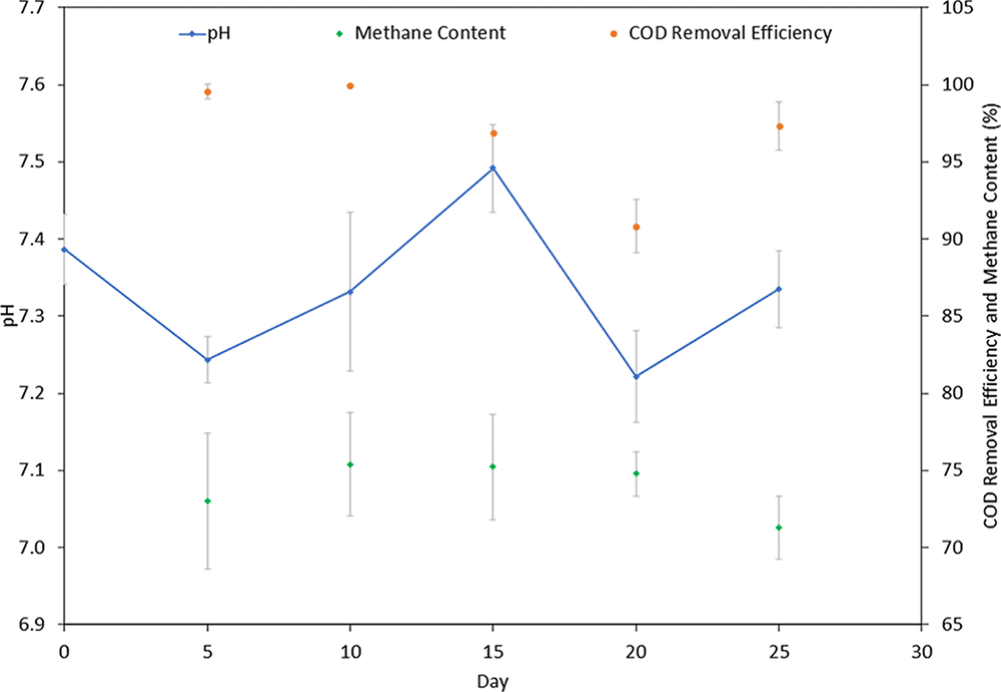
Expanded granular sludge bed (EGSB) stability and treatment efficiency.

The pH range in the LBRs for the first 20 days was in the range of 3.81–4.02; thereafter, the pH settled into the range of 4.3–4.8 (Figure 6b). A gradual rise in pH was evident in studies utilizing LBR systems in the treatment of grass, BSGs, and the organic fraction of municipal solid waste.^7,25,34^ The average pH for the EGSB was 7.3 (Figure 7). Overall, the EGSB pH remained within the range for optimum methane production in the range of 7.0–7.6.^35^

The leachate COD steadily declined from 34.8 to 27.5 g L^–1^ over the first 15 days, thereafter, values ranged between 27.7 and 32.4 g L^–1^ (Figure 6b). The initial reduction in COD can be attributed to the LBRs not being completely anaerobic; such systems are often considered partially anaerobic. It was hypothesized that aerobic respiration within the system may have converted some of the COD to carbon dioxide. These findings concur with previous findings in literature.^4,26^

The VFA concentrations (Figure 6a) in the leaching trials showed an increasing trend, reaching a peak concentration of 10,341 mg L^–1^ by day 35. Thereafter, the VFAs ranged between 8890 and 10,144 mg L^–1^. The VFAs concentration was higher than that reported in a study of VFA production from BSGs hydrolysate (8.4 g VFA L^–1^).^6^ The composition on day 35 approximated acetic acid at 20%, butyric acid at 55%, and propionic acid at 15%, with small amounts of caproic acid (6%) and other VFAs evident. Caproic acid concentrations reached 964.2 mg L^–1^ on day 45. There was a gradual rise in VFAs, due to an accumulation of longer-chain VFAs (such as isovaleric acid and valeric acid) in the EGSB from 96.7 mg VFA L^–1^ to 747.6 mg VFA L^–1^; however, this concentration is within the recommended process stability levels (<1 g VFA L^–1^).^12^

### 3.2 Trends in Treatment Efficiency

Overall, the volatile solids content of mWBM “dry” reduced over the 15 day digestion period by 20–33%. The reduction in volatile solids content was higher than in a study evaluating grass in LBRs which established a 19% decrease in volatile solids content.^7^ However, it was lower than another study which investigated the organic fraction of municipal waste in LBRs, where 40% VS destruction was reported.^15^ This evidences the difficulty or ease of degradation of specific feedstocks. The degree of acidification can be measured in two ways. First, by comparing the COD of the VFAs to the COD of mWBM “wet” (termed the COD to VFA efficiency). On day 35, when the leachate contained 10,341 mg tVFAs L^–1^ (17.3 g VFA-COD_equiv_ L^–1^) and the COD of mWBM “wet” was 33.9 g COD L^–1^, the COD to VFA efficiency was approximately 51%. Second, the degree of acidification can also be measured by comparing the COD of the VFAs to the COD within the leachate (termed acidification). On day 35, when the leachate contained 29.6 g COD L^–1^ and 17.3 g VFA-COD_equiv_ L^–1^, the acidification was approximately 58.4%. This acidification was higher than that previously reported for cheese whey (up to 40%), winery wastewater (14%), and BSGs hydrolysate (41%).^9,36,37^ As the LBRs did not contain any inoculum, it can be assessed that leaching mWBM “dry” with mWBM “wet” as leachate can extract volatile content from the solid material for VFA production.

The EGSB COD removal efficiency (Figure 7) throughout the experimental trials remained high, with an average of 96%. High COD removal efficiencies (>80%) in high-rate reactors such as the EGSB are expected. A study where a two-phase EGSB processed fermentation wastewater reported a COD removal efficiency of 95%.^24^ Another study that assessed distillery wastewater using a two-phase UASB reported a removal efficiency of 96%.^23^

### 3.3 Biogas Quality and Estimated Methane Yield

The average methane content in the biogas was high at 75%_vol_ (Figure 7). The methane content in the biogas was akin to that of a study evaluating ethanol wastewater in a two-phase UASB (75%).^38^ At peak VS destruction (33%) (see Section 3.2) and EGSB COD removal efficiency (96%), the estimated specific methane yield was 241 L CH_4_ kg^–1^ VS_mWBM_ which is 53% of the theoretical yield (459 L CH_4_ kg^–1^ VS) with the current configuration (see Box S3). This result is higher than a similar reactor degrading grass silage (204 L CH_4_ kg^–1^ VS_added_) but lower than a study degrading food waste (340 L CH_4_ kg^–1^ VS_added_).^18,19^

### 3.4 Reactor Considerations and Future Research

The LBR-EGSB reactor was shown to be capable of processing all solid (draff and cake maize) and liquid (thin stillage and centrate) distillery by-products (mWBM). The dry by-product fractions were suitable for the LBR, while the liquid by-product fractions provided suitable initial leachate. Implementing an EGSB allowed a doubling of upward velocities (0.2 m h^–1^) compared to previous UASB studies; in essence, this may reduce the required footprint of an EGSB at the distillery. The implementation of an external GLSS device to capture any residual gases produced by the effluent can potentially reduce the amount of methane slippage as compared to previous designs. The improved design of the LBR-EGSB can alleviate previous design flaws, namely, malfunctioning leachate actuation systems and low upflow velocities (0.1 m h^–1^) recommended in literature.^7,11^

Interestingly, while the LBR may not have been operated at optimum conditions, the VFA concentrations during the leaching process peaked at levels greater than 10 g VFA L^–1^. As hydrolysis is the limiting step of the performance of LBR-EGSB, a balance between VFA production, pH, and VS destruction in the LBR must be established. Increasing the overall VS destruction efficiency of the LBR would increase the overall performance of the reactor. Several options, such as thermal pretreatment, pH control, and bulk density additives, to name a few, are available and require further research.^29,39,41^ While the design of the LBR is simple, a level of ambiguity in its operation is evident in literature. Some of these variables include adding or excluding inoculum in the LBRs, flooded or percolated LBR operation and recirculation of effluent from the downstream EGSB back to the LBRs.^26,41,42^ The addition of the whiskey by-product liquids as leachate offers a unique opportunity beyond previous systems in which fresh water may be required for leaching. Overall, further research is required to establish the optimum operational conditions for the processing of both liquid and solid whiskey by-products in an LBR-EGSB.

While this study focused on the design and construction of the LBR-EGSB, the proof of concept was validated by the acidification of the leachate. A biological optimization study over a more extended period would be required to establish the optimal (maximum) specific methane potential of the whiskey by-products in the LBR-EGSB.

## 4 Conclusions

A two-phase anaerobic reactor (termed LBR-EGSB) was designed, constructed, and conceptually validated to valorize liquid and solid distillery by-products. The work presented achieved the following:

1. The LBR-EGSB was designed and constructed to process whiskey by-products.
2. The operational start-up parameters for the LBR-EGSB included a HRT of ca. 32 min for the LBR and an upflow velocity of 0.2 m h^–1^ for the expanded granular sludge bed reactor. The maximum organic loading rate was determined to be 2.6 g COD L^–1^_reactor_ d^–1^ for the expanded granular sludge bed reactor. Reactor modifications were made for sludge flotation, settling of residual solids, and pressure differential issues.
3. Phenomenological commissioning trials were conducted experimentally. The LBR-EGSB was considered commissioned once VFA concentration levels and biogas methane content were stable.
4. High VFA yields (10,341 mg L^–1^), acidification (58%), and EGSB COD removal (96%) were evident from the designed LBR-EGSB system when processing whiskey by-products. Methane-rich biogas (75%_vol_ CH_4_) was produced with an estimated yield of 241 L CH_4_ kg^–1^ VS.

## Supporting Information Available

Theoretical substrate composition calculation of mWBM; specific methane activity (SMA) of granular sludge; optimum HRT to produce VFA-rich leachate; EGSB acclimatization experimental trials; and theoretical LBR-EGSB biogas yield (PDF)

## Abbreviations/Nomenclature

AD: anaerobic digestion
BSGs: brewers spent grains
CH_4_: methane
CO_2_: carbon dioxide
COD: chemical oxygen demand
CSTR: continuously stirred tank reactor
EGSB: expanded granular sludge blanket
GLSS: gas–liquid–solid separator
HRT: hydraulic retention time
LBR: leach bed reactor
mWBM: modified whiskey by-product mixture
mWBM ‘dry&rsquo: dry fraction of mWBM: draff and cake maize
mWBM ‘wet&rsquo: wet fraction of mWBM: thin stillage and centrate
OLR: organic loading rate
TS: total solids
UASB: upflow anaerobic sludge blanket
VFAs: volatile fatty acids
VFAs-COD_equiv_: the COD equivalent of the VFAs concentration
VS: volatile solids
ww: wet weight

## References

[ref1] Yamaka W.; Phadkantha R.; Rakpho P. Economic and Energy Impacts on Greenhouse Gas Emissions: A Case Study of China and the USA. Energy Rep. 2021, 7, 240–247. 10.1016/J.EGYR.2021.06.040.

[ref2] Barrena Martínez J.; López Fernández M.; Romero Fernández P. M. Corporate Social Responsibility: Evolution through Institutional and Stakeholder Perspectives. Eur. J. Manag. Bus. Econ. 2016, 25, 8–14. 10.1016/J.REDEE.2015.11.002.

[ref3] Abad V.; Avila R.; Vicent T.; Font X. Promoting Circular Economy in the Surroundings of an Organic Fraction of Municipal Solid Waste Anaerobic Digestion Treatment Plant: Biogas Production Impact and Economic Factors. Bioresour. Technol. 2019, 283, 10–17. 10.1016/J.BIORTECH.2019.03.064. 30897388

[ref4] Nizami A.-S.; Singh A.; Murphy J. D. Design, Commissioning, and Start-Up of a Sequentially Fed Leach Bed Reactor Complete with an Upflow Anaerobic Sludge Blanket Digesting Grass Silage. Energy Fuels 2011, 25, 823–834. 10.1021/ef101739d.

[ref5] Chernicharo C. A. de L. Anaerobic Reactors; IWA Publishing, 2007.

[ref6] Castilla-Archilla J.; Heiberger J.; Mills S.; Hilbig J.; Collins G.; Lens P. N. L. Continuous Volatile Fatty Acid Production From Acid Brewery Spent Grain Leachate in Expanded Granular Sludge Bed Reactors. Front. Sustain. Food Syst. 2021, 5, 5 10.3389/fsufs.2021.664944.

[ref7] Wall D. M.; Allen E.; O’Shea R.; O’Kiely P.; Murphy J. D. Investigating Two-Phase Digestion of Grass Silage for Demand-Driven Biogas Applications: Effect of Particle Size and Rumen Fluid Addition. Renewable Energy 2016, 86, 1215–1223. 10.1016/j.renene.2015.09.049.

[ref8] Bastidas-Oyanedel J. R.; Bonk F.; Thomsen M. H.; Schmidt J. E. Dark Fermentation Biorefinery in the Present and Future (Bio)Chemical Industry. Rev. Environ. Sci. Biotechnol. 2015, 14, 473–498. 10.1007/s11157-015-9369-3.

[ref9] Atasoy M.; Owusu-Agyeman I.; Plaza E.; Cetecioglu Z. Bio-Based Volatile Fatty Acid Production and Recovery from Waste Streams: Current Status and Future Challenges. Bioresour. Technol. 2018, 268, 773–786. 10.1016/j.biortech.2018.07.042. 30030049

[ref10] Gunes B.; Stokes J.; Davis P.; Connolly C.; Lawler J. Pre-Treatments to Enhance Biogas Yield and Quality from Anaerobic Digestion of Whiskey Distillery and Brewery Wastes: A Review. Renew. Sustain. Energy Rev. 2019, 113, 109281 10.1016/j.rser.2019.109281.

[ref11] Nigam P. S. An Overview: Recycling of Solid Barley Waste Generated as a by-Product in Distillery and Brewery. Waste Manag. 2017, 62, 255–261. 10.1016/J.WASMAN.2017.02.018. 28237364

[ref12] Drosg B. Process Monitoring in Biogas Plants; IEA Bioenergy, 2013.

[ref13] López-Gutiérrez I.; Montiel-Corona V.; Calderón-Soto L. F.; Palomo-Briones R.; Méndez-Acosta H. O.; Razo-Flores E.; Ontiveros-Valencia A.; Alatriste-Mondragón F. Evaluation of the Continuous Methane Production from an Enzymatic Agave Bagasse Hydrolysate in Suspended (CSTR) and Granular Biomass Systems (UASB). Fuel 2021, 304, 121406 10.1016/j.fuel.2021.121406.

[ref14] Murphy J. D.; Thamsiriroj T. Fundamental Science and Engineering of the Anaerobic Digestion Process for Biogas Production. In The Biogas Handbook; Woodhead Publishing, 2013; pp 104–130.

[ref15] Uke M. N.; Stentiford E. Enhancement of the Anaerobic Hydrolysis and Fermentation of Municipal Solid Waste in Leachbed Reactors by Varying Flow Direction during Water Addition and Leachate Recycle. Waste Manag. 2013, 33, 1425–1433. 10.1016/J.WASMAN.2013.02.020. 23541498

[ref16] Chen T. H.; Huang J. L. Anaerobic Treatment of Poultry Mortality in a Temperature-Phased Leachbed–UASB System. Bioresour. Technol. 2006, 97, 1398–1410. 10.1016/J.BIORTECH.2005.07.002. 16112856

[ref17] Kato M. T.; Field J. A.; Versteeg P.; Lettinga G. Feasibility of Expanded Granular Sludge Bed Reactors for the Anaerobic Treatment of Low-Strength Soluble Wastewaters. Biotechnol. Bioeng. 1994, 44, 469–479. 10.1002/BIT.260440410. 18618781

[ref18] Lehtomäki A.; Huttunen S.; Lehtinen T. M.; Rintala J. A. Anaerobic Digestion of Grass Silage in Batch Leach Bed Processes for Methane Production. Bioresour. Technol. 2008, 99, 3267–3278. 10.1016/j.biortech.2007.04.072. 17702572

[ref19] Browne J. D.; Murphy J. D. The Impact of Increasing Organic Loading in Two Phase Digestion of Food Waste. Renewable Energy 2014, 71, 69–76. 10.1016/j.renene.2014.05.026.

[ref20] Linke B.; Rodríguez-Abalde Á.; Jost C.; Krieg A. Performance of a Novel Two-Phase Continuously Fed Leach Bed Reactor for Demand-Based Biogas Production from Maize Silage. Bioresour. Technol. 2015, 177, 34–40. 10.1016/j.biortech.2014.11.070. 25479391

[ref21] Akunna J. Performance of a Granular-Bed Anaerobic Baffled Reactor (GRABBR) Treating Whisky Distillery Wastewater. Bioresour. Technol. 2000, 74, 257–261. 10.1016/S0960-8524(00)00017-1.

[ref22] Yeoh B. G. Two-Phase Anaerobic Treatment of Cane-Molasses Alcohol Stillage. Water Sci. Technol. 1997, 36, 441–448. 10.2166/wst.1997.0621.

[ref23] Uzal N.; Gökçay C. F.; Demirer G. N. Sequential (Anaerobic/Aerobic) Biological Treatment of Malt Whisky Wastewater. Process Biochem. 2003, 39, 279–286. 10.1016/S0032-9592(03)00071-2.

[ref24] Wu S.; Dang Y.; Qiu B.; Liu Z.; Sun D. Effective Treatment of Fermentation Wastewater Containing High Concentration of Sulfate by Two-Stage Expanded Granular Sludge Bed Reactors. Int. Biodeterior. Biodegrad. 2015, 104, 15–20. 10.1016/J.IBIOD.2015.04.015.

[ref25] Panjičko M.; Zupančič G. D.; Zelić B. Anaerobic Biodegradation of Raw and Pre-Treated Brewery Spent Grain Utilizing Solid State Anaerobic Digestion. Acta Chim. Slov. 2015, 62, 818–827. 10.17344/acsi.2015.1534. 26680709

[ref26] Browne J. D.; Allen E.; Murphy J. D. Improving Hydrolysis of Food Waste in a Leach Bed Reactor. Waste Manag. 2013, 33, 2470–2477. 10.1016/j.wasman.2013.06.025. 23886490

[ref27] Yasar A.; Ahmad N.; Chaudhry M. N.; Khan A. A. A. Sludge Granulation and Efficiency of Phase Separator in UASB Reactor Treating Combined Industrial Effluent. J. Environ. Sci. 2007, 19, 553–558. 10.1016/S1001-0742(07)60092-8. 17915683

[ref28] Yoda M.; Nishimura S. Controlling Granular Sludge Floatation in UASB Reactors. Water Sci. Technol. 1997, 36, 165–173. 10.2166/wst.1997.0588.

[ref29] Hussain A.; Filiatrault M.; Guiot S. R. Acidogenic Digestion of Food Waste in a Thermophilic Leach Bed Reactor: Effect of PH and Leachate Recirculation Rate on Hydrolysis and Volatile Fatty Acid Production. Bioresour. Technol. 2017, 245, 1–9. 10.1016/J.BIORTECH.2017.08.130. 28892677

[ref30] Forbes C.; O’Reilly C.; McLaughlin L.; Gilleran G.; Tuohy M.; Colleran E. Application of High Rate, High Temperature Anaerobic Digestion to Fungal Thermozyme Hydrolysates from Carbohydrate Wastes. Water Res. 2009, 43, 2531–2539. 10.1016/J.WATRES.2009.03.014. 19371919

[ref31] Walter W. G. Standard Methods for the Examination of Water and Wastewater; American Public Health Association: Washington, DC, 1998.

[ref32] Kang X.; Lin R.; Li L.; Wu B.; Deng C.; O’Shea R.; Sun Y.; Murphy J. D. Assessment of Pretreatment and Digestion Temperature on Anaerobic Digestion of Whiskey Byproducts and Microbial Taxonomy. Energy Convers. Manag. 2021, 243, 114331 10.1016/j.enconman.2021.114331.

[ref33] Switzenbaum M. S.; Farrell J. B.; Pincince A. B. Relationship between the Van Kleeck and Mass-Balance Calculation of Volatile Solids Loss. Water Environ. Res. 2003, 75, 377–380. 10.2175/106143003x141187. 12934831

[ref34] Dogan E.; Dunaev T.; Erguder T. H.; Demirer G. N. Performance of Leaching Bed Reactor Converting the Organic Fraction of Municipal Solid Waste to Organic Acids and Alcohols. Chemosphere 2009, 74, 797–803. 10.1016/j.chemosphere.2008.10.028. 19042007

[ref35] Gerardi M. H. The Microbiology of Anaerobic Digesters; John Wiley & Sons, 2003.

[ref36] Guarda E. C.; Oliveira A. C.; Antunes S.; Freitas F.; Castro P. M. L.; Duque A. F.; Reis M. A. M. A Two-Stage Process for Conversion of Brewer’s Spent Grain into Volatile Fatty Acids through Acidogenic Fermentation. Appl. Sci. 2021, 11, 3222 10.3390/app11073222.

[ref37] Buitrón G.; Martínez-Valdez F. J.; Ojeda F. Biogas Production from a Highly Organic Loaded Winery Effluent Through a Two-Stage Process. BioEnergy Res. 2019, 12, 714–721. 10.1007/s12155-019-09984-7.

[ref38] Jiraprasertwong A.; Karnchanapaisal P.; Seneesrisakul K.; Rangsunvigit P.; Chavadej S. High Process Activity of a Two-Phase UASB (Upflow Anaerobic Sludge Blanket) Receiving Ethanol Wastewater: Operational Conditions in Relation to Granulation Development. Biomass Bioenergy 2021, 148, 106012 10.1016/j.biombioe.2021.106012.

[ref39] Hu Z.-H.; Yu H.-Q.; Zhu R.-F. Influence of Particle Size and PH on Anaerobic Degradation of Cellulose by Ruminal Microbes. Int. Biodeterior. Biodegrad. 2005, 55, 233–238. 10.1016/j.ibiod.2005.02.002.

[ref40] Xu S. Y.; Lam H. P.; Karthikeyan O. P.; Wong J. W. C. Optimization of Food Waste Hydrolysis in Leach Bed Coupled with Methanogenic Reactor: Effect of PH and Bulking Agent. Bioresour. Technol. 2011, 102, 3702–3708. 10.1016/j.biortech.2010.11.095. 21195606

[ref41] Xu S. Y.; Karthikeyan O. P.; Selvam A.; Wong J. W. C. Effect of Inoculum to Substrate Ratio on the Hydrolysis and Acidification of Food Waste in Leach Bed Reactor. Bioresour. Technol. 2012, 126, 425–430. 10.1016/J.BIORTECH.2011.12.059. 22227144

[ref42] Nizami A. S.; Thamsiriroj T.; Singh A.; Murphy J. D. Role of Leaching and Hydrolysis in a Two-Phase Grass Digestion System. Energy Fuels 2010, 24, 4549–4559. 10.1021/ef100677s.

