# Supplementary material for: Design, Construction, and Concept Validation of a Laboratory-Scale Two-phase Reactor to Valorize Whiskey Distillery By-products
Source: ACS Eng Au. 2023 May 13;3(4):224–34. doi: 10.1021/acsengineeringau.3c00006 (PMC10436281; doi:10.1021/acsengineeringau.3c00006)
Supplement: Supplementary file 1 — eg3c00006_si_001.pdf [file eg3c00006_si_001.pdf]

Supporting Information

The Design, Construction, and Concept Validation of a Laboratory Scale Two-Phase Reactor to Valorise Whiskey Distillery By-Products

Anga Hackula, Richard O'Shea, Jerry D. Murphy, and David M. Wall*

SFI MaREI Centre for Energy, Climate and Marine, Environmental Research Institute, University College Cork, College Road, Cork, T23 XE10, Ireland

Civil, Structural and Environmental Engineering, School of Engineering and Architecture, University College Cork, College Road, Cork, T12 K8AF, Ireland

*Email: david.wall@ucc.ie

Appendix S1: Theoretical Substrate Composition Calculation of mWBM

Box S1: mWBM Substrate Composition on a TS, VS and Total Weight Basis.

	Unit	Draff	Thin Stillage	Thick Stillage	Cake Maize	Centrate
Per Annum	(tww)	31251	322813	277503	$m_{ww_{cake}}$	$m_{ww_{cent}}$
%ww of mWBM	(%)	4.95	51.11	43.94		
Wet Ratio		1	10	9		
Total solids	(%)	27.38	2.94	8.87	29.02	3.54
TS Amount	(t TS)	8557	9500	24615		
Volatile Solids	(% TS)	95.99	88.77	95.62	97.22	89.25
Volatile Solids	(%ww)	26.28	2.61	8.48	28.21	3.16
VS Amount	(t VS)	8213	8433	23536		
VS Ratio		1	1.03	2.87		
% VS Ratio of mWBM		20	21	59		

Table S1: Whiskey by-products and characteristics used to calculate cake maize and centrate annual production

The total wet weight of thick stillage ($m_{ww_{thick}}$) must equal the combined wet weight of cake maize ($m_{ww_{cake}}$) and centrate ($m_{ww_{cent}}$). $m_{ww_{cake}}$ and $m_{ww_{cent}}$ can be rewritten with the known volatile content of the wet weight (Table S1):

$$m_{ww_{thick}} = m_{ww_{cake}} + m_{ww_{cent}}$$

$$m_{ww_{thick}} = \frac{m_{vs_{cake}}}{X_{vs_{cake}}} + \frac{m_{vs_{cent}}}{X_{vs_{cent}}}$$

Where:

$X_{vs_{cake}}$:$\%VS\ of\ cake\ maize$

$X_{vs_{cent}}$:$\%VS\ of\ centrate$

$$277503\ t = \frac{m_{vs_{cake}}}{0.2821} + \frac{m_{vs_{cent}}}{0.0316}$$

- Multiply by 0.2821

$$78284 = m_{vs_{cake}} + \frac{2821}{316} m_{vs_{cent}}$$

$$m_{vs_{cake}} = 78284 - \frac{2821}{316} m_{vs_{cent}} \quad \text{- } \textit{equation 1}$$

The VS of thick stillage ($m_{vs_{thick}}$) must equal the combined VS of Cake Maize ($m_{vs_{cake}}$) and Centrate($m_{vs_{cent}}$).

$$m_{vs_{thick}} = m_{vs_{cake}} + m_{vs_{cent}}$$

$$23536\ t = m_{vs_{cake}} + m_{vs_{cent}}$$

$$m_{vs_{cake}} = 23536 - m_{vs_{cent}} \text{ - } \textit{equation 2}$$

Substitute equation 1 into 2:

$$78284 - \frac{2821}{316} m_{vs_{cent}} = 23536 - m_{vs_{cent}}$$

$$78284 - 23536 = (\frac{2821}{316} - 1) m_{vs_{cent}}$$

$$m_{vs_{cent}} = 6906\ t\ VS\ per\ annum$$

$$\therefore m_{ww_{cent}} = \frac{m_{vs_{cent}}}{0.0316} = \frac{6906}{0.0316} = 218544\ t\ WW\ per\ annum$$

Substitute $m_{vs_{cent}}$ into equation 1:

$$m_{vs_{cake}} = 23536 - m_{vs_{cent}} = 23536 - 6906 = 16630\ t\ VS\ per\ annum$$

$$\therefore m_{ww_{cake}} = \frac{m_{vs_{cake}}}{0.2821} = \frac{16630}{0.2821} = 58951\ t\ WW\ per\ annum$$

The annual total solids can be calculated from the wet weight and the %TS from Table 1:

$$m_{ts_{cake}} = m_{ww_{cake}} \times 0.2902 = 58951 \times 0.2902 = 17108\ t\ TS\ per\ annum$$

$$m_{ts_{cent}} = m_{ww_{cent}} \times 0.0354 = 218544 \times 0.0354 = 7736\ t\ TS\ per\ annum$$

The percentage composition of mWBM on a wet weight basis and volatile solid basis can be calculated using the annual production rate.

$$\% \, Centrate_{VS\,basis} = \frac{m_{vs_{cent}}}{m_{vs_{draff}} + m_{vs_{thin}} + m_{vs_{cake}} + m_{vs_{cent}}} = \frac{6906}{8213 + 8433 + 16630 + 6906} = 17.2\%$$

Centrate VS in mWBM

$$\% \, Centrate_{ww\,basis} = \frac{m_{ww_{cent}}}{m_{ww_{draff}} + m_{ww_{thin}} + m_{ww_{cake}} + m_{ww_{cent}}}$$

$$\% \, Centrate_{ww\,basis} = \frac{218544}{31251 + 322813 + 58951 + 218544} = 34.6\%$$

The same method was used to calculate the percentage of cake maize produced per annum. Table S1 Shows the VS and WW composition and theoretical quantities of the substrates that make up mWBM

	Draff	Thin Stillage	Cake Maize	Centrate
**VS Basis (% VS mWBM)**	20	21	41	17
**VS Mass (t VS per annum)**	8213	8433	16630	6906
**WW Basis (% WW mWBM)**	5	51	9	35
**Wet Weight (t WW per annum)**	31251	322813	58951	218544

Table S2: mWBM composition on a VS and WW basis.

The theoretical VS content of mWBM can be calculated from Table S2:

$$\%VS = \frac{m_{VS} \, of \, substrates}{m_{ww} \, of \, substrates} = \frac{8213 + 8433 + 16630 + 6906}{31251 + 322813 + 58951 + 218544}$$

$$\%VS = 6.36 \approx \mathbf{6.4 \, \%VS \, in \, mWBM}$$

The theoretical TS content of mWBM was calculated similarly.

Appendix S2: Specific Methane Activity (SMA) of Granular Sludge

The specific methane activity (SMA) test is used to establish the methane-producing capacity of the inoculum. The SMA provides insight into the reactor's organic loading rate, particularly during an anaerobic reactor's start-up (Nizami et al., 2011). The SMA was determined by adding a known amount of volatile solids content in the inoculum (1.61 gVS) into a glass bottle and a known amount of acetic acid (2.2 g COD. L^{-1}) (Hussain & Dubey, 2017). The gas production was monitored during this period using the Bioprocess Control ™ AMPTS II. The bottles were kept at 37°C in a water bath. The SMA is approximated by plotting the g COD equivalent of methane production against time (in hours) and dividing it by the gVS.

SMA = methane produced (g COD-CH_4). g^{-1}VS

Equations B.1 and B.2 are required to convert methane to the COD equivalent (Chernicharo, 2007):

$K(t) = (P.K)/[R.(273 + T)]$ – Equation B.1

$V_{CH4} \times K(t) = Methane\ produced\ in\ sCOD\ equivalent\ (gCH_4 - COD)$ – Equation B.2

Where:

K(t) = correctional factor due to reactor operating temperature (g COD.L_{CH4}^{-1})

P = atmospheric pressure (1 atm)

K = One mole of CH_4 in COD (64 g COD per mole)

R = gas constant (0.08206 atm.L/mole.°K)

T = temperature of the reactor (°C)

V_{CH4}= volume of methane produced (L)

Box S2: Specific Methane Activity (SMA) of Granular Sludge Used in EGSBs.

The correction factor was calculated using equation B.1

$$K(t) = \frac{P.K}{[R.(273 + T)]} = \frac{1 \times 64}{[0.08206 \times (273 + 37)]} = 2.52 \text{ g COD. } L_{CH_4}^{-1}$$

Figure S1 shows the cumulative methane production.

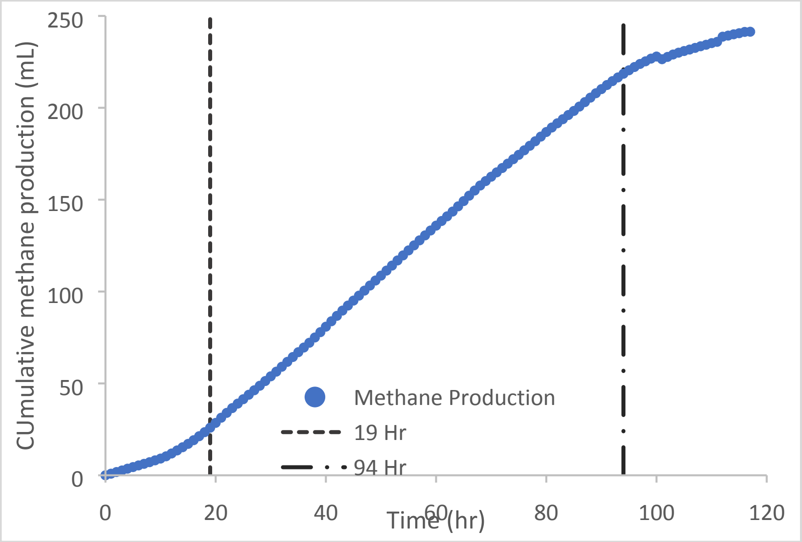

Figure S1: Cumulative methane production from the SMA test.

The SMA was estimated by using the gradient at the steepest region (Chernicharo, 2007) in Figure S1, which is between 19-94 hrs. The corresponding change in methane in this region is 192.63 L CH_4 (218.53-25.90 L CH_4). The hourly rate during this 75-hr period (94-19hrs) equates to 2.57 L CH_4. hr^{-1} or 61.64 L CH_4. d^{-1}.

Using the correction factor (K(t) = 2.52 g COD. $L_{CH_4}^{-1}$) the COD equivalent of methane produced is 155.33 mg COD-CH_4. d^{-1}. Using the volatile solids introduced in the SMA assay (1.61 gVS), the SMA of the inoculum is ± **0.1 g COD-CH_4. g^{-1}VS. d^{-1}**. Several studies have shown SMAs above 0.6 g COD. g^{-1}VS. d^{-1} (Nizami et al., 2011). This SMA is low, but studies have suggested that it could increase as inert particles tend to float and exit the reactor system (Hussain and Dubey, 2017a). A study evaluating UASBs that treated raw domestic sewage had a low SMA value (0.01 gCOD-CH_4. g^{-1}VS. d^{-1}) that increased over time to 0.1 gCOD-CH_4. g^{-1}VS. d^{-1} due to the long start-up time (22 weeks) (Kalogo et al., 2001). Thus, it is expected that the start-up of the EGSBs using this sludge may take a long time.

Approximately 367ml of acetic acid (2.2 g COD. L^{-1}) was used in the SMA test, which equates to 807.4 mg COD. Using Equations B.1 and B.2, it can be shown that the theoretical maximum gas production from acetic acid is 320.40 L CH_4 (807.4 mg COD /2.52 g COD. $L_{CH_4}^{-1}$). The actual gas production from the SMA test was 241.4 L CH_4. Therefore, the conversion efficiency of the inoculum is **±75 %.**

Appendix S3: Optimum HRT to Produce VFA-Rich Leachate

The HRT is the average duration the liquid fraction remains within a reactor. The dilution rate (Φ) is the volumetric flow rate (Q) that enters the working volume (V_e) and is the inverse of the HRT for LBRs. A high dilution rate, which equates to a low HRT, is desired. However, a dilution rate that is too high may result in flooding of the substrate and/or washing out of essential microbes. Flooding and microbial washout may negatively impact the leaching process; therefore, a balance must be established. The HRT for LBRs can be calculated as per Eq.C.1 (Browne et al., 2013).

$$HRT = V_e/Q \qquad \textbf{Equation C.1}$$

Where: HRT is the hydraulic retention time, V_e is the working volume of the reactor, and Q is the volumetric flow rate of the LBR influent.

To establish the optimum HRT that produces VFA-rich leachate, three different configurations were evaluated. Each LBR was loaded with the solid fraction and liquid fraction of mWBM; 0.67kg of mWBM ‘dry’ was added to each LBR, and 4.02kg of mWBM ‘wet’ was loaded into three separate 5L leachate holding tanks. To reflect low, medium and high recirculation rates, 60, 100 and 140 $L.day^{-1}$ were chosen, achieving HRTs of approximately 72 minutes, 43 minutes and 31 minutes, respectively. The EGSBs were disconnected during this period. Previous studies by Nizami et al. (2010), Wall et al. (2015) and Browne et al. (2013) operated at an HRT of approximately 3.8hrs, 4.4hrs and 3.8hrs respectively, for a 16L (working volume) LBR system. However, a study found that HRTs lower than 9hrs lead to pooling on the substrate surface, but this is dependent on the reactor configuration and the substrate. The leaching trial was operated in pooling/flooding conditions. Figure S2 shows the VFA composition during the preliminary leaching trials.

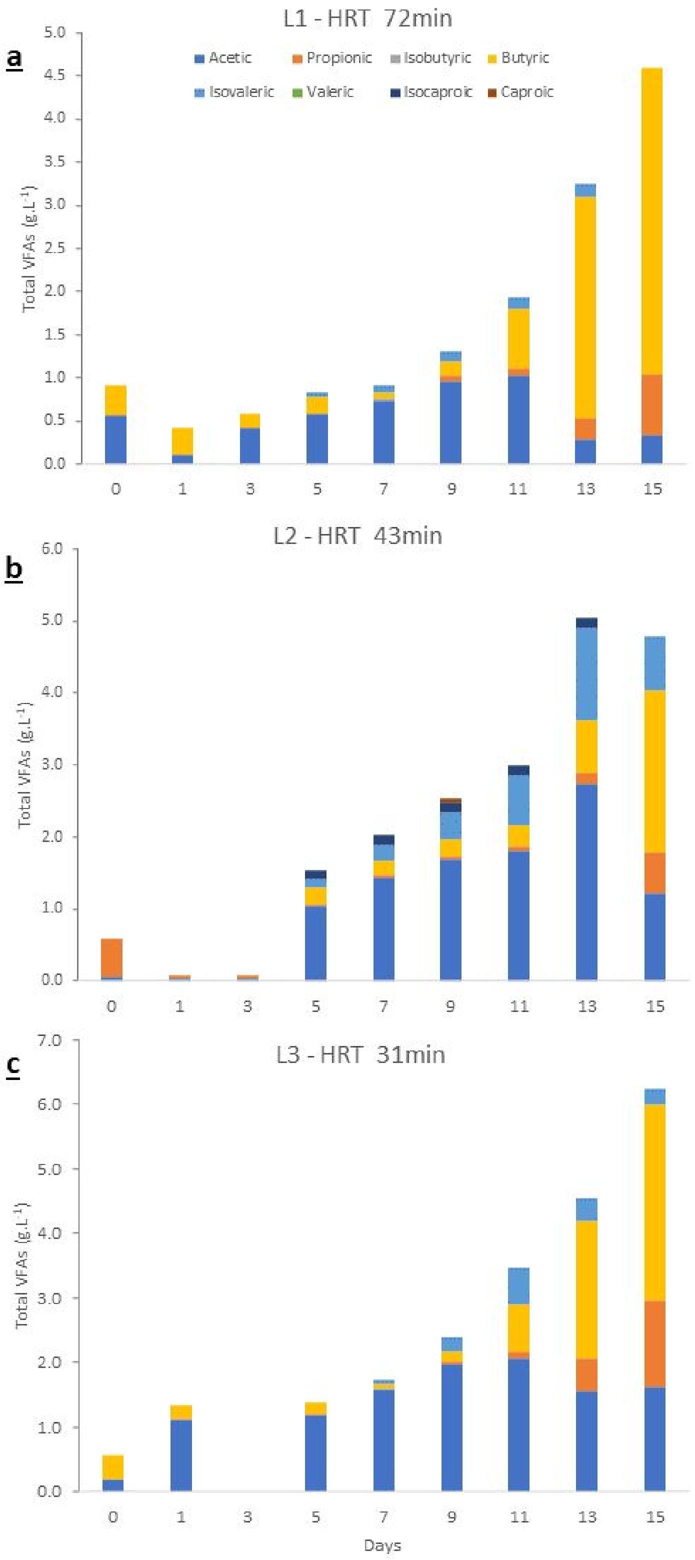

Figure S2: VFA profiles of short leaching trials.

The lowest HRT (31 min) yielded the highest VFA yield but resulted in pooling within the LBR. As VFA yield was the primary objective, the 31 min HRT, 140 L.d^{-1} recirculation rate, was used for the commissioning of the LBRs.

Appendix S4: EGSB Acclimatisation Experimental Trials

The EGSBs were acclimatized through experimental trials and operated over two distinct phases over 82 days as follows (Figure S3):

- Phase 1 (Day 0-56): the EGSB was fed directly with raw mWBM ‘wet’ independently of the LBRs. This was done to acclimatize the inoculum to mWBM ‘wet’ and allow inactive sludge to be washed out from the EGSB. Initially, the OLR was 0.7 g COD. L^{-1}. d^{-1} (HRT of 23 days) and gradually increased to a maximum OLR of ±2.6 g COD. L^{-1}. d^{-1} (HRT of 6 days).
- Phase 2 (Day 57-82): the EGSB was fed VFA-rich leachate generated from the LBR preliminary trials (see Appendix S3) but was still disconnected from the LBRs. This was done to acclimatize the inoculum to the VFA-rich leachate. The OLR started at 2.2 g COD. L^{-1}. d^{-1} (HRT of 6 days) for 22 days and increased to ±2.6 g COD. L^{-1}. d^{-1} (HRT of 6 days). While the OLR changed, the HRT remained the same due to the increased COD concentration of the VFA-rich leachate.
- Phase 3 (Day 83-112): the EGSB was connected directly to the LBR leachate after the LBR leaching trials. The EGSB influent flow rate was the same as the settings on day 82. As the COD of the leachate varied, the average COD over the previous 7 days was used to set the OLR to ±2.6 g COD. L^{-1}. d^{-1} (HRT of 6 days) for the following week.

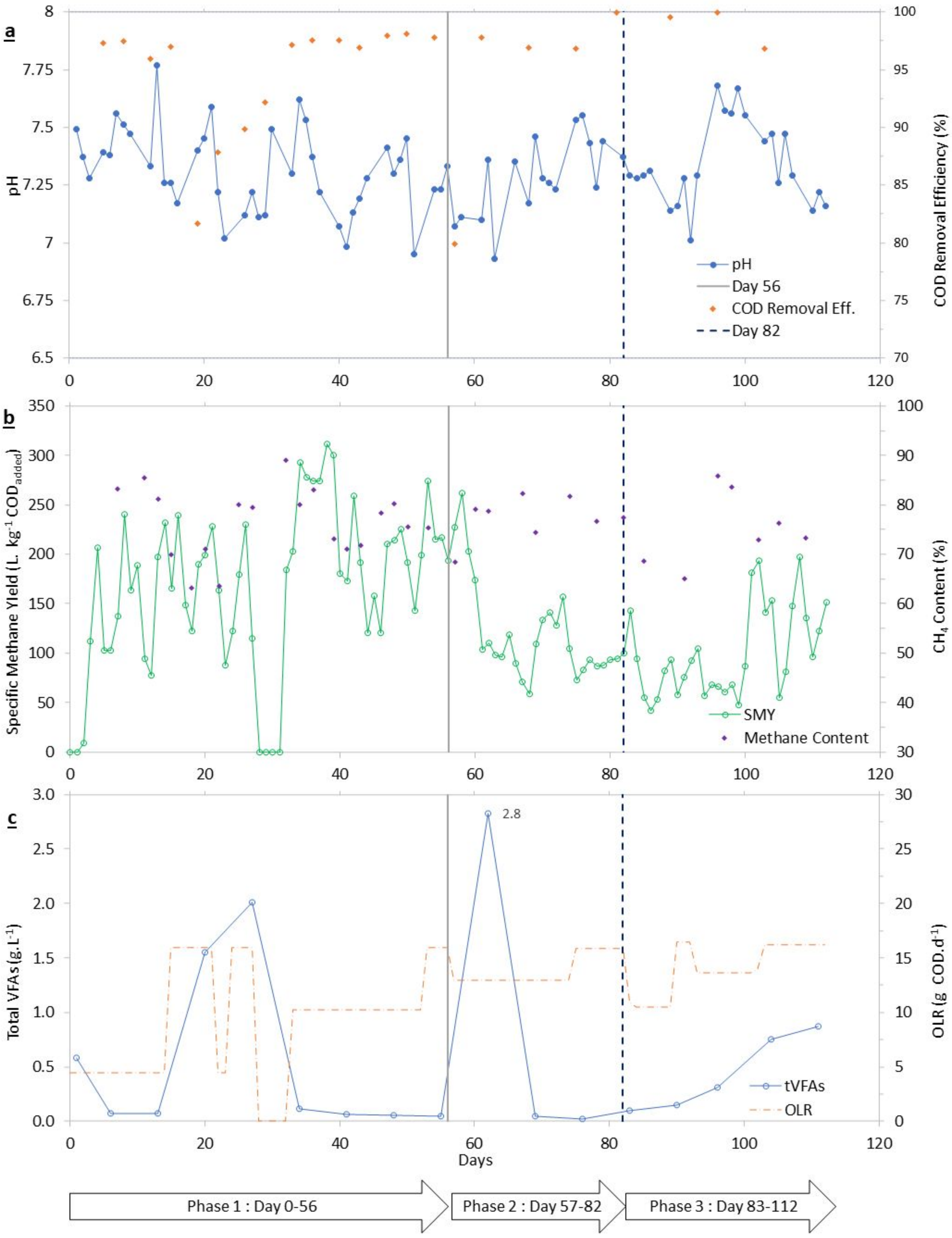

Figure S3: (a) Expanded granular sludge bed (EGSB) pH and COD removal efficiencies, (b) specific methane yield and biogas methane content, (c) EGSB effluent total VFAs and the organic loading rate.

Appendix S5: Theoretical LBR-EGSB Biogas Yield

The theoretical biogas, methane, and COD yield for the treatment of mWBM in the LBR-EGSB is shown in Box S3

Box S3: Estimation of COD, Biogas and CH_4 Production in the LBR-EGSB (adapted from (Nizami et al., 2011).)

±4.67 kg of mWBM (4.00 kg mWBM 'wet' + 0.67 kg mWBM 'dry') was loaded into the reactor sequentially every 5 days. The solid retention time (SRT) of mWBM 'dry' was therefore 15 days (3 LBRs x 5 days).

I. Breakdown of volatile matter in 3 LBRs

mWBM 'dry' in 3 LBRs	3 x 0.67 kg mWBM 'dry'	=	2.01	kg mWBM 'dry'
mWBM 'dry' %TS at	28.9% – see Table 1	=	581	g of TS
VS (% of TS) at	96.3%	=	560	g of VS
VS destruction	33%	=	185	g of VS
Solid retention time (SRT)		=	21	days

II. COD Production from mWBM

It can be shown that 1 g of VS produces 1.4 g of COD. The COD production from mWBM 'dry' introduced to the LBRs can be calculated as follows:

VS-to-COD conversion	=	1.4	g COD g^{-1} VS
VS conversion at 70%	=	185	g VS x 1.4 g COD g^{-1}VS (see **I.**)
**Total COD from mWBM dry**	=	**259**	**g COD from mWBM 'dry'**

The leachate COD consists of the COD originating in mWBM 'wet' and the COD generated from mWBM 'dry'. mWBM 'wet' consists of centrate (±40%) and thin stillage (±60%). Therefore, the total COD produced after 1 SRT (15 days) can be calculated as follows:

Thin stillage added every 7 days = 60 %ww of mWBM 'wet'= 2.4 kg (4kg x 0.6) ≈ 2.4 L
Thin stillage added after 15 days = 3.5 L x 3 = 7.2L
Total COD from thin stillage added = 7.2 L x 33.4 g COD. L^{-1} of thin stillage (see Table 1) = 240 g COD
A similar calculation for the COD contributed by centrate = 168 g COD

Total COD from mWBM 'wet' = 240 g COD (from thin stillage) + 168 g COD (from centrate)
= 408 g COD

The total COD of mWBM is the sum of the COD of mWBM 'wet' and mWBM 'dry'.
Total COD in mWBM = 240 g COD mWBM 'dry' + 408 g COD mWBM 'wet'
= **648** **g COD mWBM** after 21 days

III. Biogas Production from mWBM

1 kg of COD destroyed generates 350 L of CH_4. The methane production can be estimated from the COD production and efficiency of the EGSB (95%) (Chernicharo. 2007). The methane production from the estimated COD produced from the LBR is as follows:

∴ 350 L CH_4 kg^{-1} COD x 648 g COD x 95% efficiency
Methane produced after 21 days = 215 L CH_4

The theoretical specific methane yield of mWBM in the LBR-EGSB can be estimated by using the amount of VS introduced into the system after 3 feeds of mWBM and the methane produced after 21 days:

Theoretical VS introduced = 4.67 kg of mWBM x 3 Feeds x VS of mWBM (see Box S1)
Theoretical VS introduced = 4.67 kg x 3 x 6.37% = 892 g VS mWBM introduced after 21 days.

Theoretical methane yield for LBR-EGSB = 215 L CH_4 / 892 g VS mWBM
Theoretical methane yield for LBR-EGSB **= 241 L CH_4. kg^{-1} VS**
Theoretical methane yield for LBR-EGSB = **172 L CH_4. kg^{-1} COD** (using 1.4 kg COD. kg^{-1}VS)

The maximum yield **(459 L CH_4. kg^{-1} VS)** was calculated assuming 100% VS destruction and 100% COD removal efficiency.

Appendix References

1. Nizami, A.-S., Singh, A., & Murphy, J. D. (2011). Design, Commissioning, and Start-Up of a Sequentially Fed Leach Bed Reactor Complete with an Upflow Anaerobic Sludge Blanket Digesting Grass Silage. *Energy & Fuels*, *25*(2), 823–834. https://doi.org/10.1021/ef101739d
2. Hussain, A., & Dubey, S. K. (2017). Specific methanogenic activity test for anaerobic degradation of influents. *Applied Water Science*, *7*(2), 535–542. https://doi.org/10.1007/S13201-015-0305-Z/TABLES/2
3. Chernicharo, C. A. de L. (2007). *Anaerobic Reactors* (Vol. 3). IWA Publishing. https://www.iwapublishing.com/sites/default/files/ebooks/9781780402116.pdf
4. Kalogo, Y., MBouche, J. H., & Verstraete, W. (2001). Physical and Biological Performance of Self-Inoculated UASB Reactor Treating Raw Domestic Sewage. *Journal of Environmental Engineering*, *127*(2), 179–183. https://doi.org/10.1061/(ASCE)0733-9372(2001)127:2(179)
5. Browne, J. D., Allen, E., & Murphy, J. D. (2013). Improving hydrolysis of food waste in a leach bed reactor. *Waste Management*, *33*(11), 2470–2477. https://doi.org/10.1016/j.wasman.2013.06.025
6. Wall, D. M., Allen, E., O'Shea, R., O'Kiely, P., & Murphy, J. D. (2016). Investigating two-phase digestion of grass silage for demand-driven biogas applications: Effect of particle size and rumen fluid addition. *Renewable Energy*, *86*, 1215–1223. https://doi.org/10.1016/j.renene.2015.09.049
